# Gene and Cell Therapy for Epilepsy: A Mini Review

**DOI:** 10.3389/fnmol.2022.868531

**Published:** 2022-05-11

**Authors:** Alisa A. Shaimardanova, Daria S. Chulpanova, Aysilu I. Mullagulova, Zaid Afawi, Rimma G. Gamirova, Valeriya V. Solovyeva, Albert A. Rizvanov

**Affiliations:** ^1^Institute of Fundamental Medicine and Biology, Kazan Federal University, Kazan, Russia; ^2^Center for Neuroscience, Ben Gurion University of the Negev, Be’er Sheva, Israel

**Keywords:** epilepsy, gene therapy, cell therapy, adeno-associated virus, mesenchymal stem cells, neural stem cells, mononuclear cells, encapsulated cell biodelivery

## Abstract

Epilepsy is a chronic non-infectious disease of the brain, characterized primarily by recurrent unprovoked seizures, defined as an episode of disturbance of motor, sensory, autonomic, or mental functions resulting from excessive neuronal discharge. Despite the advances in the treatment achieved with the use of antiepileptic drugs and other non-pharmacological therapies, about 30% of patients suffer from uncontrolled seizures. This review summarizes the currently available methods of gene and cell therapy for epilepsy and discusses the development of these approaches. Currently, gene therapy for epilepsy is predominantly adeno-associated virus (AAV)-mediated delivery of genes encoding neuro-modulatory peptides, neurotrophic factors, enzymes, and potassium channels. Cell therapy for epilepsy is represented by the transplantation of several types of cells such as mesenchymal stem cells (MSCs), bone marrow mononuclear cells, neural stem cells, and MSC-derived exosomes. Another approach is encapsulated cell biodelivery, which is the transplantation of genetically modified cells placed in capsules and secreting various therapeutic agents. The use of gene and cell therapy approaches can significantly improve the condition of patient with epilepsy. Therefore, preclinical, and clinical studies have been actively conducted in recent years to prove the benefits and safety of these strategies.

## Introduction

Epilepsy is one of the most common diseases of the nervous system (more than 50 million cases have been reported worldwide). This condition is characterized by recurrent unprovoked seizures that result from abnormally excessive firing of neurons due to an imbalance in levels of excitation and inhibition in the brain (Stafstrom and Carmant, 2015). Epilepsy can have a variety of etiologies: structural, infectious, metabolic, immune, genetic as well as unknown (Perucca et al., 2020). Despite the active research in this area, the causes of the disease are still unclear. Epilepsy is also a group of conditions that are heterogeneous in manifestations and causes, making it difficult to develop unambiguous diagnostic criteria (Thijs et al., 2019). Patients suffer from seizures that often worsen over time and is accompanied by cognitive function deterioration and mental health problems. Patients often become resistant to existing antiepileptic drugs (Sheng et al., 2018).

The production of effective methods of treatment is an urgent problem that requires an immediate solution since epilepsy is a serious medical and social problem. The risk of premature death in people with epilepsy is three times higher than in the general population (according to WHO^1^).

Currently, gene and cell therapy is being investigated as a way to reduce neuronal loss, inflammation, oxidative stress, and the frequency and duration of epileptic seizures. The new approaches being developed are capable of increasing the survival of neurons, improving neurogenesis, providing neuroprotection and preserving cognitive functions.

**FIGURE 1:**
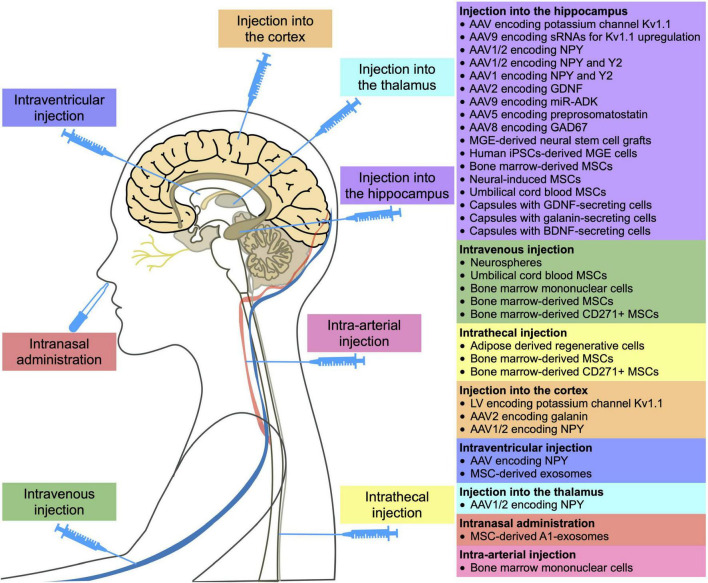
Methods of administration of gene and cell drugs for epilepsy reported in *in vivo* and clinical studies.

Thus, this paper discusses the promising results of gene and cell therapy for epilepsy. Table 1 and Figure 1 provide detailed information on existing *in vivo* studies and clinical trials.

**TABLE 1:** Gene and cell therapy for epilepsy.

Therapeutic agent	Model	Dose and duration	Therapy results	References
**GENE THERAPY**				
LV encoding potassium channel Kv1.1	Rat model of tetanus toxin- induced epilepsy	Single injection of 1–1.5 mL of LV [2.6 × 10^9^ viral genomes (vg/mL)] into layer 5 of the right motor cortex	Decrease in the frequency of seizures over several weeks	Wykes et al., 2012
AAV encoding potassium channel Kv1.1	Rat model of kainic acid-induced status epilepticus	Single injection of 8.0 μl of AAV (8.3 × 10^14^ vg/mL) into dorsal and ventral hippocampus	Decrease in the frequency and duration of seizures	Snowball et al., 2019
AAV9 encoding small guide RNAs for Kv1.1 upregulation	Mouse model of kainic acid-induced status epilepticus	Single injection of 300 nL of AAV9 (8 × 10^12^ vg/ml) into right ventral hippocampus	Decrease in the frequency of spontaneous generalized seizures	Colasante et al., 2020
AAV1/2 encoding NPY	Rat model of kainate-induced seizures	Single injection of 2 μl of AAV2 (10^12^ vg/mL) into dorsal hippocampus	Decrease in the frequency of kainate-induced seizures	Gotzsche et al., 2012
AAV encoding NPY	Rat model of kainate-induced seizures	Single injection of 10 μL of AAV (5 × 10^11^ vg/mL) into the right lateral ventricle	Decrease in the frequency of kainate-induced seizures	Dong et al., 2013; Zhang et al., 2013
AAV1/2 encoding NPY and Y2	Rat model of kainate-induced seizures	Single injection of 1 μl of AAV-NPY (10^12^ vg/mL) and 1.5 μl of AAV-Y2 (10^12^ vg/mL) into dorsal and ventral hippocampus	Decrease in the frequency of kainate-induced seizures	Ledri et al., 2016
AAV1/2 encoding NPY	Rat model of genetic generalized epilepsy	Single injection of 3 μl of AAV (6.6 × 10^12^ vg/ml) into thalamus and 1 μl into SC	Decrease in the frequency and duration of seizures in the thalamus, decrease in the frequency of seizures in the SC	Powell et al., 2018
AAV1 encoding NPY and Y2	Rat model of kainate-induced seizures	Single injection of 3 μL of AAV (10^12^ vg/mL) into hippocampus	Decrease in the frequency of seizures	Melin et al., 2019
AAV2 encoding GDNF	Rat model of kindling-induced epilepsy	Single injection of 1 and 2 μl of virus (2.1 × 10^12^ vg/mL) into dorsal and ventral hippocampus	Decrease in the frequency of seizures, increase in seizure induction threshold	Kanter-Schlifke et al., 2007
AAV9 encoding miR-ADK	Rat model of kainate-induced seizures	Single injection of 3 μL of AAV (9.48 × 10^11^ vg/mL) vector infused into hippocampus	Decrease in the frequency of seizures, protection of dentate hilar neurons	Young et al., 2014
AAV5 encoding preprosomatostatin	Rat model of kindling-induced epilepsy	Single injection of 2 μL of AAV (4.19 × 10^13^ vg/mL) into the left and right CA1 region and dentate gyrus	Development of seizure resistance in 50% of animals	Natarajan et al., 2017
AAV8 encoding GAD67	The EL/Suz mouse model of epilepsy	Single injection of 3 μL of AAV (1 × 10^10^ vg/mL) bilaterally into the CA3 region of hippocampus	Significant reduction in seizure generation	Shimazaki et al., 2019
AAV2 encoding galanin	Rat model of kainate-induced seizures	Single injection of 2 μl of AAV (8 × 10^12^ vg/mL) into the piriform cortex	Prevention of kainic acid-induced seizures	McCown, 2006
**CELL THERAPY**				
**Nervous system cells**				
Intravenous infusion of neurospheres	Rat model of pilocarpine-induced status epilepticus	Single intravenous injection of 2 × 10^6^ cells	Decrease in the oxidative stress damage	de Gois da Silva et al., 2018
Transplantation of medial ganglionic eminence-derived neural stem cell grafts	Rat model of kainic acid-induced status epilepticus	Single transplantation of 4 grafts of 80,000 cells in each side of the hippocampus (640,000 cells/rat)	Suppression of spontaneous recurrent motor seizures, an increase in the number of GABAergic neurons, restoration of GDNF expression. No improvement in cognitive function	Waldau et al., 2010
GABAergic interneuron precursors from the medial ganglionic eminence	Kv1.1 mutant mice	Bilateral transplantation into the deep layers of the cortex at two different sites on the hemisphere (4 × 10^5^ cells/mouse)	Decrease in the duration and frequency of spontaneous electrographic seizures	Baraban et al., 2009
Human iPSCs-derived medial ganglionic eminence cells	Mouse model of pilocarpine-induced status epilepticus	Transplantation of cells in the hippocampus (3 × 10^5^ cells/mouse)	Suppression of seizures, aggressiveness, hyperactivity, improvement of cognitive function	Cunningham et al., 2014
Human iPSCs-derived medial ganglionic eminence cells	Rat model of kainic acid-induced status epilepticus	Single transplantation of 3 grafts of 100,000 cells in each side of the hippocampus (600,000 cells/rat)	Relief of spontaneous recurrent seizures, improvement of cognitive function and memory, reduction in the loss of interneurons	Upadhya et al., 2019
**MSCs**				
Undifferentiated autologous bone marrow-derived MSCs (in combination with anti-epileptic drugs)	Patients with epilepsy	Single intravenous injection of 1–1.5 × 10^6^ cells/kg and single intrathecal injection of 1 × 10^5^ cells/kg after 5–7 days	No serious side effects. Reduction in frequency or complete stop of seizures, improvement of clinical manifestations	Hlebokazov et al., 2017; Hlebokazov et al., 2021 NCT02497443
Adipose derived regenerative cells	Patients with autoimmune refractory epilepsy	Intrathecal injection of 4 ml of stromal fraction, 3 times every 3 months	Complete remission in 1 of 6 patients (within 3 years), mild and short-term reduction in seizure (3 of 6 patients). Improvement in patients’ daily functioning. No further regression was observed for 3 years	Szczepanik et al., 2020 NCT03676569
Bone marrow-derived CD271^+^ MSCs and bone marrow MSCs	Pediatric patients with drug-resistant epilepsy	Combination therapy consisting of single intrathecal (0.5 × 10^9^) and intravenous (0.38 × 10^9^–1.72 × 10^9^) injections of bone marrow-derived CD271^+^ MSC and four intrathecal injections of bone marrow MSC (18.5 × 10^6^–40 × 10^6^) every 3 months	Neurological and cognitive improvement, decrease in the frequency of seizures	Milczarek et al., 2018
Bone marrow-derived MSCs	Rat model of pilocarpine- induced status epilepticus	Single intravenous injection of 3 × 10^6^ cells/rat	Decrease in the frequency of seizures, increase in the number of neurons	Abdanipour et al., 2011
Bone marrow-derived MSCs	Rat model of lithium-pilocarpine- induced epilepsy	Single intravenous injection of 10^6^ cells/rat	Inhibition of epileptogenesis and improvement of cognitive functions	Fukumura et al., 2018
Bone marrow-derived MSCs	Rat model of pilocarpine-induced status epilepticus	Single injection of 100,000 cells in each side of the hippocampus (200,000 cells/rat) or single intravenous injection of 3 × 10^6^ cells/rat	Reduction of oxidative stress in the hippocampus, decrease in the levels of inflammatory cytokines (TNF-α and IL-1β) and an apoptotic marker (caspase 3). Improvement of neurochemical and pathological changes in the brain	Salem et al., 2018
Neural-induced adipose-derived stem cells	Rat model of kainic acid-induced status epilepticus	Single transplantation into the hippocampus (50,000 cells/rat)	Decrease in seizure activity, recovery of memory and learning ability	Wang et al., 2021
Umbilical cord blood MSCs	Rat model of pentylenetetrazole-induced chronic epilepsy	Single intravenous injection (10^6^ cells/rat)	Decrease in the severity of seizures and oxidative stress damage, improved motor coordination and cognitive function	Mohammed et al., 2014
Umbilical cord blood MSCs	Rat model of lithium-pilocarpine induced status epilepticus	Single transplantation into the hippocampus (5 × 10^5^ cells/rat)	Partial restoration of glucose metabolism in the hippocampus, seizure frequency did not differ from the control group	Park et al., 2015
**Exosomes**				
MSC-derived exosomes	Mouse model of pilocarpine-induced status epilepticus	Single intraventricular injection (30 μg)	Reduction in the intensity of manifestation of reactive astrogliosis and inflammatory response, improvement in cognitive functions and memory	Xian et al., 2019
MSC-derived A1-exosomes	Mouse model of pilocarpine-induced status epilepticus	Two intranasal administrations after 18 h (15 μg)	Reduction in the loss of glutamatergic and GABAergic neurons, reduction in the inflammation, support of normal hippocampal neurogenesis, cognitive function, and memory	Long et al., 2017
**Mononuclear cells**				
Bone marrow mononuclear cells	Patients with temporal lobe epilepsy	Single intra-arterial injection (1.52–10 × 10^8^ cells/patient)	Decrease in the number of seizures, increase in average memory indicators. Complete disappearance of seizures in 40% of patients	DaCosta et al., 2018 NCT00916266
Bone marrow mononuclear cells	Rat model of lithium-pilocarpine induced status epilepticus	Single intravenous injection (1 × 10^6^ cells/rat)	Prevention of spontaneous recurrent seizures in the early stage of epilepsy, a significant reduction in the frequency and duration of seizures in the chronic phase	Costa-Ferro et al., 2010
Bone marrow mononuclear cells	Mouse model of pilocarpine- induced status epilepticus	Single intravenous injection (2 × 10^6^ cells/mouse)	Neuroprotective and anti-inflammatory effects, decrease in the loss of hippocampal neurons	Leal et al., 2014
**Encapsulated cell biodelivery**				
Semipermeable capsule containing GDNF-secreting ARPE-19 cells (arising retinal pigment epithelia cells)	Rat model of pilocarpine- induced status epilepticus	Transplantation in the hippocampus (5 × 10^4^ cells/capsule). GDNF concentration up to 566.79 ± 192.47 ng/24 h	Decrease in the frequency of seizures, cognitive function improvement, neuroprotective effect	Paolone et al., 2019
Semipermeable capsule containing GDNF-secreting cells	Rat model of kindling-induced epilepsy	Transplantation in the hippocampus. High GDNF concentration –150.92 ± 44.51 ng/ml/24 h, low concentration – 0.04 ± 0.88 ng/ml/24 h	Low GDNF levels have an antiepileptic effect compared to elevated levels	Kanter-Schlifke et al., 2009
Semipermeable capsule containing galanin-secreting ARPE-19 cells	Rat model of kindling-induced epilepsy	Transplantation in the hippocampus (6 × 10^4^ cells/capsule) High galanin concentration – 12.6 ng/ml/24 h, low concentration – 8.3 ng/ml/24 h	High doses decrease the duration of focal seizures	Nikitidou et al., 2014
Semipermeable capsule containing BDNF-secreting ARPE-19 cells	Rat model of pilocarpine- induced status epilepticus	Transplantation in the hippocampus (5 × 10^4^ cells/capsule). BDNF concentration – 200–400 ng/24 h	Decrease in the frequency of seizures. Cognitive function improvement	Falcicchia et al., 2018
Semipermeable capsule containing BDNF-secreting baby hamster kidney cells	Rat model of pilocarpine- induced status epilepticus	Transplantation in the hippocampus (10^6^ cells/capsule) BDNF concentration – 7.2 ± 1.2 ng/24 h	Injection of low doses had a neuroprotective effect and stimulated neurogenesis	Kuramoto et al., 2011

## Gene Therapy

The current focus of gene therapy strategies for epilepsy is primarily aimed at reducing neuronal excitability by overexpressing neuro-modulatory peptides such as neuropeptide Y (Dong et al., 2013; Zhang et al., 2013), galanin etc. (McCown, 2006) or by the genetic modification of astrocytes, for example, to suppress adenosine kinase (ADK) expression (Young et al., 2014).

Overexpression of ion channels such as the Kv1 potassium channel, which reduces the intrinsic excitability of neurons, increases the threshold for action potential generation required for neuron firing. Thus, permanent inhibition of the increased internal excitability of neurons inside the epileptic focus may have a long-term antiepileptic effect (Snowball et al., 2019). In a rat model of chronic refractory focal neocortical epilepsy, a lentivirus encoding Kv1.1 was shown to suppress epileptic activity for several weeks upon injection into an epileptic focus (Wykes et al., 2012). In addition, the use of adeno-associated virus (AAV) overexpressing Kv1.1 has resulted in a decrease in both the frequency and duration of seizures in the temporal lobe epilepsy (TLE) model (Snowball et al., 2019). CRISPRa-mediated Kv1.1 upregulation decreased spontaneous generalized seizures in the mouse model of TLE (Colasante et al., 2020).

Another approach in antiepileptic treatment is the suppression of the activity of the excitatory mediator glutamate. Neuropeptide Y (NPY) preferentially binds to three G-protein coupled receptors (Y1, Y2, and Y5). Furthermore, the antiepileptic effect of NPY in the hippocampus is mediated by binding of NPY with presynaptic Y2 or Y5 receptors, which subsequently suppress glutamate release at excitatory synapses (Berglund et al., 2003). Binding of NPY to Y1 receptors leads to an increase in the epileptic activity (Benmaamar et al., 2003). Thus, genetic modification of the hippocampus by viruses with NPY can reduce the frequency of spontaneous seizures by 40% in both the TLE model (Noe et al., 2008) and kainate-induced seizures model (Dong et al., 2013; Zhang et al., 2013). Additionally, AAV-NPY was shown to have therapeutic effects when injected into the thalamus or somatosensory cortex (SC) of genetic epilepsy model rats (Powell et al., 2018). In addition to viruses encoding the NPY, viruses encoding the receptors for this protein have also been administered to suppress seizures, which significantly increased the antiepileptic effect. For example, the injection of AAV1/2 with the NPY and Y5 significantly reduced the number of kainate-induced seizures (Gotzsche et al., 2012). The use of AAV1/2 viral vector encoding the NPY in combination with Y2 resulted in the reduction in the number of kainate-induced seizures by 31–45% (Ledri et al., 2016; Melin et al., 2019). The mechanisms of the antiepileptic effect of NPY are still unclear. However, overexpression of NPY is known to result in the expression of the N-methyl-D-aspartate receptor subunits NR1, NR2A, and NR2B, which plays a critical role in the development of epilepsy (Dong et al., 2013). Moreover, the level of the synaptophysin protein, whose expression increased in the hippocampus of patients with epilepsy, was also decreased in the kainate-induced seizures model (Zhang et al., 2013). Overexpression of NPY has been studied in limited studies. Some studies have shown no effect on short- or long-term memory (Szczygiel et al., 2020), while others have illustrated that NPY decreases synaptic plasticity and negatively affects hippocampal-based spatial discrimination learning (Sorensen et al., 2008).

In addition to the treatment approaches described above, AAVs are actively being used to deliver proteins with therapeutic properties. Nevertheless, these are primarily *in vivo* studies that have not yet been further developed. For example, the introduction of AAV encoding the glial cell line-derived neurotrophic factor (GDNF) gene or the γ-aminobutyric acid decarboxylase 67 (GAD67) gene reduced the seizure frequency in the TLE model (Kanter-Schlifke et al., 2007; Shimazaki et al., 2019). Moreover, promising results were demonstrated for AVVs encoding preprosomatostatin injected into the hippocampus (Natarajan et al., 2017).

Astrocyte transduction using AAV9 differs from the approach based on genetic modification of neurons described above. For example, miR-mediated suppression of ADK expression in astrocytes allowed an increase in the concentration of adenosine, which plays a pivotal role in seizure termination, and led to a decrease in the duration of kainate-induced seizures (Young et al., 2014).

A potential limitation of the viral vectors for the treatment of epilepsy is that viruses can transduce not only the epileptic focus cells, but also surrounding brain areas, so it can be difficult to determine the optimal dosage to achieve a therapeutic effect without impairing normal brain function. These difficulties can be overcome by a new strategy for creating a group of G-protein coupled receptors called designer receptors exclusively activated by designer drugs (DREADDs). DREADD technology is based on mutated human muscarinic receptors, which, when expressed in cells, can only be specifically bound and activated by the pharmacologically inert compounds, such as clozapine-N-oxide (CNO). Systemic administration of CNO leads to the opening of Gi-protein gated inwardly rectifying potassium channels, resulting in membrane hyperpolarization and neuronal inhibition. The efficacy of DREADDs has already been confirmed in a number of therapeutically relevant animal models of epilepsy (Lieb et al., 2019). For example, injection of AAV5 expressing a synthetic receptor hM4Di into the dorsal hippocampus of rats with pilocarpine-induced epilepsy led to a significantly reduction of seizures (Katzel et al., 2014). Another limiting factor is the high invasiveness of virus injection methods. In addition, even the most non-immunogenic AAV-based vectors are capable of inducing both humoral and cellular immune responses, which can greatly influence the outcome of gene therapy, since neutralizing antibodies can bind the viral particles and significantly reduce the transduction efficiency (Ronzitti et al., 2020). Some viral vectors can also lead to insertional mutagenesis due to genome integration (Athanasopoulos et al., 2017). In addition, the capacity of the vector is of great importance. For example, AAVs can only package ∼4.7 kb of DNA (Weinberg and McCown, 2013).

In gene therapy for epilepsy, the use of AAVs encoding potassium channel or NPY genes are the most investigated. However, the evaluation of the effectiveness of these therapeutic agents has not gone beyond *in vivo* animal systems. To date, there are no registered clinical trials aimed at investigating gene drugs for the treatment of epilepsy. In this regard, we can say that gene therapy is at an early stage of development and obviously requires more attention from researchers.

## Cell Therapy

Cell therapy for epilepsy includes a variety of approaches, including the use of MSCs (Abdanipour et al., 2011; Mohammed et al., 2014; Park et al., 2015; Hlebokazov et al., 2017, 2021; Fukumura et al., 2018; Salem et al., 2018; Melin et al., 2019; Szczepanik et al., 2020; Wang et al., 2021), mononuclear cells (Costa-Ferro et al., 2010; Leal et al., 2014; DaCosta et al., 2018), cells of the nervous system (Baraban et al., 2009; Waldau et al., 2010; Cunningham et al., 2014; de Gois da Silva et al., 2018; Upadhya et al., 2019), and encapsulated cells expressing various therapeutic factors (Kanter-Schlifke et al., 2009; Kuramoto et al., 2011; Nikitidou et al., 2014; Falcicchia et al., 2018; Paolone et al., 2019), as well as exosomes (Long et al., 2017; Xian et al., 2019). Cell therapy is an alternative treatment that can be used to reduce the incidence rate of seizures in epilepsy, including drug-resistant epilepsy, as shown in several clinical studies (Hlebokazov et al., 2017, 2021; DaCosta et al., 2018; Milczarek et al., 2018; Szczepanik et al., 2020).

### Transplantation of Neural Cells

Promising approaches for epilepsy treatment are aimed at replenishing the neuronal loss in the hippocampus by transplanting cells of the nervous system. The neural stem cell is capable of self-renewal and differentiation into neurons, glial cells, and oligodendrocytes as well as expression of neurotrophic factors. Transplantation of neural stem cells into the hippocampus of epileptic rats led to an increase in the number of GABAergic neurons and cells expressing GDNF, which resulted in the suppression of seizures (Waldau et al., 2010).

The protective role of neurospheres, which are spherical clusters of neural stem cells grown *in vitro*, have also been studied. Intravenous administration of neurospheres into epileptic rats has been shown to reduce oxidative damage by significantly increasing the level of antioxidant enzymes such as glutathione, superoxide dismutase and catalase (de Gois da Silva et al., 2018).

It has been shown that progenitor cells from embryonic medial ganglionic eminence (MGE) can differentiate into functional GABAergic interneurons. Transplantation of MGE precursors into the cortex of model mice with a deletion of potassium channels led to a significant reduction in the duration and frequency of spontaneous electrographic seizures, which is achieved due to GABA-mediated synaptic inhibition (GABA is an inhibitory neurotransmitter) (Baraban et al., 2009).

GABAergic interneurons can be derived from induced pluripotent stem cells (iPSCs). It has been shown that transplantation of pluripotent stem cell-derived GABAergic interneurons into the hippocampus of model animals led to a decrease in seizures and other behavioral abnormalities (Cunningham et al., 2014; Upadhya et al., 2019).

### Mesenchymal Stem Cell Transplantation

Mesenchymal stem cells (MSCs) have many therapeutic properties useful in epilepsy, including neuroprotection, immunomodulation, neurogenesis support, inflammation, and oxidative stress damage suppression. These effects are achieved due to the fact that MSCs secrete various neurotrophic factors, anti-inflammatory cytokines, growth factors and other biologically active molecules (Vizoso et al., 2017; Milczarek et al., 2018). It is also known that MSCs can cross the blood-brain barrier and migrate to the affected area. Even after intravenous administration, MSCs migrate into the hippocampus of model animals with epilepsy and have a therapeutic effect (Abdanipour et al., 2011).

Numerous studies have shown that the administration of undifferentiated MSCs can significantly decrease the frequency of seizures (Hlebokazov et al., 2017, 2021; Milczarek et al., 2018; Szczepanik et al., 2020), improve cognitive (Fukumura et al., 2018; Milczarek et al., 2018; Wang et al., 2021) and motor functions (Mohammed et al., 2014), increase the number of neurons (Abdanipour et al., 2011), reduce oxidative stress (Salem et al., 2018). The introduction of MSCs promotes the survival of GABAergic interneurons (Mohammed et al., 2014; Fukumura et al., 2018).

It has been shown that MSCs begin to secrete BDNF, NT3, and NT4 after neuronal differentiation. Their transplantation into the hippocampus of rats with kainic acid-induced epilepsy led to an increase in the level of BCL-2 and BCL-XL anti-apoptotic proteins and a decrease in the level of BAX pro-apoptotic protein in the hippocampus. Suppression of seizure activity and restoration of learning ability have also been noted (Wang et al., 2021).

The results of clinical studies also confirm benefits of MSC transplantation. For example, phase I/II clinical trials have demonstrated that the administration of antiepileptic drugs along with one or two intravenous and intrathecal administrations of MSCs has been safe and effective in treating patients with drug-resistant epilepsy. Combined use of levetiracetam (an antiepileptic drug) and MSCs led to a decrease in the frequency of seizures, and a repeated course of MSC administration contributed to a further improvement in the patients’ condition (NCT02497443) (Hlebokazov et al., 2017, 2021).

A combination therapy has also been used for pediatric patients with drug-resistant epilepsy. The intrathecal and intravenous administration of autologous bone marrow nucleated cells, followed by four intrathecal injections of MSCs every 3 months, resulted in neurological and cognitive improvement in all patients, including a decrease in the number of seizures (Milczarek et al., 2018).

Intrathecal administration of adipose-derived regenerative cells (a heterogeneous population of cells, including multipotent stem cells, fibroblasts, regulatory T-cells, and macrophages) into patients with autoimmune refractory epilepsy (3 times every 3 months) has also been reported. Only 1 out of 6 patients achieved complete remission (there were no seizures for more than 3 years), in 3 out of 6 there was a slight and short-term decrease in the frequency of seizures, and in two out of six no effect was found (Szczepanik et al., 2020). In all these clinical studies, some improvement after the introduction of cells without serious side effects was observed.

### Exosome Injection

Not only MSCs, but also exosomes derived from them, exhibit immunoregulatory, anti-inflammatory and trophic effects (Harrell et al., 2019; Ha et al., 2020; Xunian and Kalluri, 2020), which means they also have great potential for the treatment of nervous systems diseases (Gorabi et al., 2019; Guy and Offen, 2020). As a result of intraventricular injection of exosomes, hippocampal astrocytes are able to take up the exosomes and attenuate astrogliosis and inflammation in model mice (Xian et al., 2019). After intranasal administration, exosomes also were able to reach the hippocampus, reduce the loss of glutamatergic and GABAergic neurons, and significantly reduce inflammation in the hippocampus of model animals (Long et al., 2017).

### Mononuclear Cell Transplantation

Bone marrow mononuclear cells (BMCs) also possess immunomodulatory and neuroprotective properties (Yoshihara et al., 2007; Shrestha et al., 2014; do Prado-Lima et al., 2019; Wei et al., 2020) due to the secretion of various trophic factors and cytokines (Liu et al., 2004; Wei et al., 2020).

It has been shown that after intravenous administration to animals with status epilepticus, BMCs can migrate to the hippocampus, reduce the frequency and duration of seizures, maintain neuronal density (Costa-Ferro et al., 2010), increase proliferation of neurons, reduce the level of inflammatory cytokines and increase the level of anti-inflammatory cytokines (Leal et al., 2014).

Phase I/II clinical trials have investigated the safety and efficacy of intra-arterial injection of BMCs in TLE patients. Researchers demonstrated safety, improved memory, a decrease in theta activity, and a decrease in spike density (DaCosta et al., 2018).

## Encapsulated Cell Biodelivery

Despite the proven efficacy, gene and cell therapy methods have a number of known disadvantages and side effects. It should be noted that these methods of therapy are irreversible and unregulated, and some of them can lead to an immune response and malignant transformation.

In the case of cell therapy, as mentioned above for gene therapy, the major limiting factor is safety, specifically the possibility of a significant immune response induction and oncogenic transformation. In some cases, long-term immunosuppressive therapy and monitoring of the cells after the administration are required (Aly, 2020). It is important to select a suitable donor in the case of allogeneic transplantation, not only in terms of HLA compatibility, but also in the heterogeneity of effector molecules secreted by the cells, which vary considerably between different donors and can lead to a significant variation in the treatment efficacy (Montzka et al., 2009; Mukhamedshina et al., 2019). The migration ability of cells also imposes restrictions on the use of cell-based therapy in clinical practice since highly invasive cell delivery is sometimes required to reach the target area and cross the blood-brain barrier (Issa et al., 2020).

One of the approaches to overcome some of the difficulties of cell-based therapy is encapsulated cell biodelivery (ECB). ECB usually involves the implantation of a capsule with a semipermeable membrane containing genetically modified cells that secrete therapeutic substances. In this case, the cells do not leave the capsule, but the therapeutically active molecules leave and spread in the area of transplantation. This method allows (1) preventing an immune response and transplant rejection, since the cells are physically isolated, (2) locally delivering therapeutic substances, and (3) stopping treatment by removing the capsule from the brain. To suppress epileptic activity, such capsules were designed to contain cells expressing GDNF (Kanter-Schlifke et al., 2009; Paolone et al., 2019), BDNF (Kuramoto et al., 2011; Falcicchia et al., 2018), or galanin (Nikitidou et al., 2014). ECB has shown positive results in epilepsy therapy since these neurotrophic factors and neuropeptide exhibit antiepileptic activity.

## Conclusion

This review discussed recent developments in gene and cell therapy for epilepsy. To date, *in vivo* models have shown the potential benefit of viral vectors (mainly AAVs, rarely LVs) encoding genes of therapeutic agents. Thus, the effectiveness of viral delivery of (1) neuro-modulatory peptides, such as NPY or galanin, (2) potassium channels to inhibit increased internal excitability of neurons, (3) GDNF, (4) GAD67, (5) preprosomatostatin, as well as (6) miR to suppress ADK expression has been shown.

Cell-based therapy has made more progress with documented clinical trials showing the benefits and safety of MSC or BMC transplantation. The therapeutic effect can be achieved due to the neuroprotective, anti-inflammatory, immunomodulatory properties of these cells, which is also shown for MSC-derived exosomes. Researchers are also focusing on the transplantation of neural stem cells into the hippocampus to reduce neuronal loss in animal models. ECB, which is the injection of capsules with cells expressing BDNF, GDNF or galanin in the hippocampus, has shown an antiepileptic effect.

The majority of gene and cell therapies have not yet reached clinical practice, but they have made significant progress. To date, preclinical studies in animal model are ongoing and new clinical trials are being registered to confirm both the effectiveness and safety of these approaches. Considering the heterogeneous nature of the onset and manifestation of epilepsy, the development of methods of gene and cell therapy can make a significant contribution to progress in the treatment of this disease.

## Author Contributions

AS, DC, VS, and AR: conceptualization. AS and DC: writing-original draft preparation. VS, AM, RG, ZA, and AR: writing-review and editing. AS: visualization. VS and AR: supervision. All authors have read and agreed to the published version of the manuscript.

## Conflict of Interest

The authors declare that the research was conducted in the absence of any commercial or financial relationships that could be construed as a potential conflict of interest.

## Publisher’s Note

All claims expressed in this article are solely those of the authors and do not necessarily represent those of their affiliated organizations, or those of the publisher, the editors and the reviewers. Any product that may be evaluated in this article, or claim that may be made by its manufacturer, is not guaranteed or endorsed by the publisher.

## Footnotes

1
https://www.who.int/ru
